# The prognosis benefits of adjuvant versus salvage radiotherapy for patients after radical prostatectomy with adverse pathological features: a systematic review and meta-analysis

**DOI:** 10.1186/s13014-019-1384-z

**Published:** 2019-11-09

**Authors:** Ronggui Tao, Jindong Dai, Yunjin Bai, Jiyu Yang, Guangxi Sun, Xingming Zhang, Jinge Zhao, Hao Zeng, Pengfei Shen

**Affiliations:** Department of Urology, Institute of Urology, and National Clinical Research Center for Geriatrics, West China Hospital, Sichuan University, No. 37 Guoxue Xiang, Chengdu, 610041 China

**Keywords:** Radical prostatectomy, Adverse pathological features, Adjuvant radiotherapy, Salvage radiotherapy, Prognosis

## Abstract

**Background:**

The appropriate timing of radiotherapy (RT) for patients after radical prostatectomy (RP) with adverse pathological features (APFs) remains controversial. This systematic review was conducted to compare the efficacy of adjuvant radiotherapy (ART) and salvage radiotherapy (SRT).

**Methods:**

PubMed, EMBASE, Web of Science and the Cochrane Library electronic databases were searched to retrieve the required. The hazard ratio (HR) and corresponding 95% confidence interval (CI) of overall survival (OS), biochemical recurrence-free survival (BRFS) and distant metastases-free survival (DMFS) were extracted. The survival benefits of ART with SRT (including early salvage radiotherapy (ESRT)) were analyzed. The process of the meta-analysis was performed with RevMan version 5.3.

**Results:**

A total of fifteen retrospective studies were finally included in the final analysis including 5586 patients. The pooled analysis indicated that ART could achieve better control of prostate cancer and improve OS (*p* = 0.0006), BRFS (*p* < 0.0001) and DMFS (p < 0.0001), when compared to SRT. The subgroup analysis of the 5-year OS rate demonstrated that the ART group still had survival advantages compared to the SRT group (*p* = 0.0006). However, ART and SRT were comparable in 10-year OS rate (*p* = 0.07). ART had advantages over SRT in both 5-year (*p* = 0.0003) and 10-year BRFS (p = 0.0003). The subgroup analysis with different follow-up starting points from RP or RT was essentially consistent with the above results. The pooled analysis also showed that ART was superior to ESRT on OS (*p* = 0.008) and DMFS (*p* = 0.03), and comparable to ESRT on BRFS (*p* = 0.1).

**Conclusions:**

According to this meta-analysis, ART could be served as a preferential treatment for patients with APFs after RP to improve prognosis. Certainly, high-quality, multicenter randomized controlled trials (RCTs) are expecting to confirm the outcomes of our meta-analysis in the future.

## Background

Radical prostatectomy (RP) or radiotherapy (RT) plus androgen-deprivation therapy (ADT) is recommended as standard treatment options for patients with high-risk localized prostate cancer (PCa) [1]. About 15–25% localized PCa patients underwent RP would develop a biochemical recurrence (BCR); and tumor recurrence of high-risk PCa after RP has always been a concern of clinicians [2]. To achieve better prognosis, RT is always recommended for patients with adverse pathological features (APFs) after RP, including extracapsular extension, seminal vesicle invasion, positive surgical margins (PSM), and high Gleason score (GS). Emerging evidence also indicates that postoperative RT could significantly control the local recurrence of tumor and reduce the risk of distant metastasis [3].

According to the timing and circumstances of the procedures, postoperative RT is divided into adjuvant radiotherapy (ART) and salvage radiotherapy (SRT). ART is given to patients with high risk of recurrence and an undetectable prostate-specific antigen (PSA) after prostatectomy due to APFs prior to recurrence. However, SRT is administrated to patients with an undetectable PSA that becomes subsequently detectable and increases on 2 measurements or a PSA that remains persistently detectable after RP [4, 5].

Even though several randomized controlled trials (RCTs) have previously indicated that patients with APFs received ART could achieve a better biochemical recurrence-free survival (BRFS) when compared to initial observation [6–8], the results from the National Cancer Data Base showed that the number of patients with APFs received post-prostatectomy ART was declining [9]. Concerns about the toxicity of radiotherapy, the tendency to choose salvage treatment after BCR and patient preference might explain this phenomenon [9]. Up to now, there is still no determined consensus on the pros and cons of these two therapies. As the optimal timing of postoperative RT remains controversial, we conducted this systematic review and meta-analysis to elevate the efficacy and the prognosis benefits of ART and SRT.

## Methods

### Search strategy

The search strategy was initiated by two reviewers respectively. To retrieve the required literature as completely as possible, a great number of databases have been searched, included PubMed (from 1950 to June 2019), EMBASE (using Ovid as the main search engine from 1974 to June 2019), Web of Science (from 1900 to June 2019) and the Cochrane Library electronic databases (from 1948 to June 2019). Combinations of the following MeSH and keywords were used in databases: (prostate neoplasms or prostate cancer or prostatic cancer) and (adjuvant radiotherapy or adjuvant RT) and (salvage radiotherapy or salvage RT).

### Inclusion and exclusion criteria

The criteria for included studies were: (1) all potential studies concerning the comparison of the prognosis of postoperative ART and SRT; (2) articles published in English; (3) at least one of the following outcomes was reported: overall survival (OS), BRFS and distant metastases-free survival (DMFS).

All patients must undergo RP and had at least one of APFs, including PSM, extracapsular extension, seminal vesicle invasion, and high GS. The specific eligibility criteria for the ART group were as follows: (1) postoperative RT was initiated when serum PSA was undetectable; (2) RT should be performed within 6 months after RP. Correspondingly, the SRT group should meet the following conditions: (1) RT was started when serum PSA rising constantly from undetectable level; (2) patients have been shown to develop PSA recurrence.

Conference abstracts which did not provide enough information were excluded. Case reports, review articles and editorial comments were not in our consideration. Neoadjuvant therapy should not be administered to these patients. The patient had other malignancies other than prostate cancer should be also ruled out.

### Data extraction and quality assessment

Two authors carried out the procedure of data extraction independently. The titles and abstracts of articles retrieved by the proposed strategy firstly were screened to rule out irrelevant articles. Then, the full texts of selected articles were elevated in complying with the inclusion and exclusion criteria. The necessary data of the finally included articles were extracted, included the type of study, authors, publication year, the characteristics of participants in the ART and SRT groups (number, age, GS, staging, and follow-up time), outcomes (OS, BRFS, DMFS and related hazard ratio (HR)), etc.

The Newcastle-Ottawa scale (NOS), included three items: Selection, Comparability, and Outcome, was used to elevate the methodological quality of each study [10]. Each article was scored on a scale of 0 to 9. A study that achieved a score of 8 or 9 was considered high quality and a score of 5 to 7 were regarded as moderate quality [11]. Discussion and consultation assisted in resolving an existed disagreement between two authors during the procedure.

### Outcomes of interest

The primary outcomes were OS and BRFS. The secondary outcome was DMFS. OS was defined as the time from RP/RT to death, irrespective of the reason of death. BRFS was calculated from RP/RT to a detectable PSA value, or a serum PSA > 0.2 ng/ml on two consecutive detections for post-RP patients, or a rise of PSA > 0.2 ng/ml above nadir for post-RT patients. DMFS was identified as the time from RP/RT to evidence of local recurrence or distant metastasis from imaging examination.

### Data synthesis and analysis

The Review Manager software (RevMan version 5.3, The Cochrane Collaboration 2014) was used to analyze the data. Two reviewers input the data and performed the analysis. The other reviewers verified it in order to minimize the chance of error and bias.

As OS, BRFS and DMFS were time-to-event outcomes and were most appropriately analyzed using HR [12], we used HR between two survival distributions as a summary statistic. For a study which reported HR and corresponding 95% confidence interval (CI), we extracted it directly. We also calculated these HRs and the corresponding 95% CIs of the included studies which provided sufficient data using the methods outlined by Tierney and colleagues [12]. In accordance with the contract, an overall HR of less than 1 favored the ART group. The survival beneficial effect of ART compared to SRT was considered statistically significant if the 95% CI of HR did not overlap 1 [13]. The reported odds ratio (OR) with 95% CI was also calculated in the analysis.

The heterogeneity among the studies was assessed using the Chi-squared test and the I^2^ statistic. A *p*-value of < 0.1 and an I^2^ value > 50% were considered as statistical heterogeneity. If significant heterogeneity was indicated, a random-effect model was used; instead, a fixed-effect model was used. Funnel plots would be used to investigate publication bias if enough studies were available.

5-year and 10-year OS and BRFS rates of these included studies were further extracted and a subgroup analysis was conducted in terms of the starting point of follow-up time. We also separately compared the survival benefits of ART with early salvage radiotherapy (ESRT), which was defined as RT administered at a postoperative serum PSA ≤ 0.5 ng/ml.

## Results

### Characteristics of included studies

**Fig. 1:**
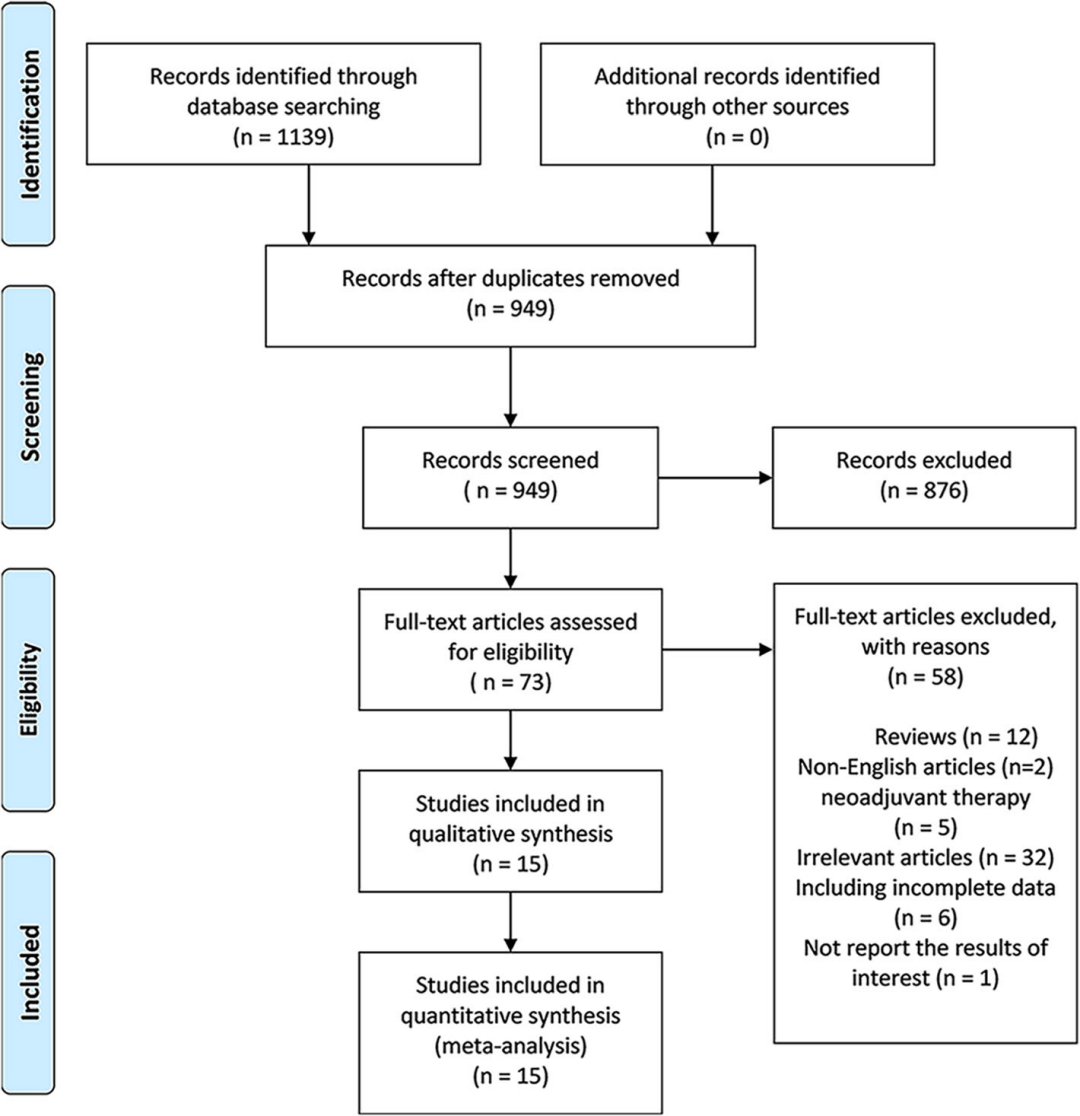
Flow diagram of qualified studies

According to our search strategy, 1139 articles were screened. A total of sixteen studies met with the predefined inclusion criteria; however, fifteen studies were finally included in the meta-analysis [14–28]. One study was excluded due to lack of necessary data, failure to report the results of interest, and unsuccessful HR extract [29]. Five studies were excluded because some subjects received neoadjuvant therapy [30–34]. Since two clinical trials paid more attention to the comparison of ART and wait-and-see, but the percentage of patients who received SRT in the observation group was very small, they were both not in our consideration [7, 35]. The selection process of qualified studies was shown in Fig. 1.

All included articles were cohort studies published between 2002 and 2018. Seven studies took RP as the starting point of follow-up and reported corresponding results [14–18, 21, 22]. Six studies followed RT as the basis of follow-up and reported related results [19, 20, 23, 24, 27, 28]. The remaining two studies reported respectively results at different follow-up starting points [25, 26]. It was worth mentioning that four articles compared the prognosis of postoperative ART and ESRT [14–17]. The detailed description of these studies was shown in Table 1.

**Table 1:** Characteristics of included studies

Reference	Study type	Country	Study period	Sample size (n)			Follow-up time (months)	
				Total	ART	SRT	ART	SRT
Briganti 2012	match-controlled	Italy	1991–2007	780	390	390	Median (IQR):71.9 (39–103)	Median (IQR):41 (10–60)
Buscariollo 2017	retrospective cohort	United States	1992–2013	401	171	230	Median (IQR):89 (55–158)	Median (IQR):96 (63–130)
Fossati 2016	retrospective cohort	Italy	1996–2009	510	243	267	Median (IQR):94 (53–126)	Median (IQR):92 (70–136)
Hwang 2018	retrospective cohort	United States	1987–2013	732	366	366	Median (IQR):65.8 (40–107)	Median (IQR):73.3 (44.9–106.6)
Borghetti 2017	retrospective cohort	Italy	1999–2012	258	127	131	Overall median:50.7	–
Hervas 2017	retrospective cohort	Spain	1991–2011	702	223	479	Overall median (range):34 (3–141)	–
Mishra 2015	retrospective cohort	United States	1990–2009	186	74	112	Overall median (range):103 (30–247)	–
Hsu 2015	retrospective cohort	United States	1995–2009	305	76	229	Overall median (range):74 (7–256)	–
Tilki 2016	retrospective cohort	Germany	2005–2013	718	213	505	Overall median (IQR):33.8 (17.1–49.0)	–
Detti 2012	retrospective cohort	Italy	1995–2010	307	203	104	Mean ± SD:3.3 ± 2.3	Mean ± SD:4.5 ± 2.5
Ost 2011	match-controlled	Belgium	1999–2009	178	89	89	Median (range):36 (3–120)	Median (range):36 (3–120)
Trabulsi 2008	matched-Control	United States	1987–2002	192	96	96	Median (range):97 (30–207)	Median (range):94 (26–190)
Tsien 2003	retrospective cohort	United States	1986–1997	95	38	57	Median (range):10.1 (4.8–14.5)	Median (range):8.8 (2.0–17.0)
Taylor 2003	retrospective cohort	United States	1988–1998	146	75	71	Median:68	Median:39

ART: adjuvant radiotherapy; SRT: salvage radiotherapy; IQR: interquartile range; SD: standard deviation

### Characteristics of patients

A total of 5586 patients with APFs after RP were enrolled in this meta-analysis, including 2419 patients received ART and 3167 patients got SRT. Of those patients received SRT, 1253 patients specifically were stated that they were treated with ESRT. The age of subjects ranged from 59 to 66 years old in different studies. The PSM rate of patients in the ART group ranged from 50 to 96%, while it ranged from 23 to 87% in the SRT group. Detailed information of included patients was summarized in Table 2.

**Table 2:** Characteristic of all included patients

Reference	Group	Age	Gleason score n, (≤6/7/≥8)	Pathologic T stage (n)	Preoperative PSA (ng/ml)	Pre-RT PSA (ng/ml)	PSM n (%)	Radiation dose (Gy)
Briganti 2012	ART	Median (IQR):64 (60–68)	160/185/45	T3a:261 T3b:129	Median(IQR): 10 (6.7–16.1)	Median(IQR): 0 (0–0)	245 (62.8)	Median(IQR): 65 (60–70)
	ESRT	Median (IQR):65 (61–69)	163/173/54	T3a:274 T3b:116	Median(IQR): 10 (6.3–14.7)	Median(IQR): 0.2 (0.1–0.3)	238 (61.0)	Median(IQR): 66 (66–66)
Buscariollo 2017	ART	Median (IQR):60 (54–65)	31/83/56	T2:43 T3:128	Median(IQR): 6 (5–10)	< 0.1	143 (83.6)	Median(IQR): 61.2 (61.2, 64.8)
	ESRT	Median (IQR):59 (54–63)	43/142/45	T2:90 T3:140	Median(IQR): 6 (5–10)	Median(IQR): 0.3 (0.2–0.4)	163 (70.9)	Median(IQR): 64.8 (64.8, 64.8)
Fossati 2016	ART	Median (IQR):64 (61–69)	57/120/66	T3a:137 ≥ T3b:106	Median(IQR): 9.3 (6.2–15.8)	Median(IQR): 0 (0–0)	181 (74)	Median(IQR): 60 (60–65)
	ESRT	Median (IQR):65 (60–70)	49/147/71	T3a:168 ≥ T3b:99	Median(IQR): 9.8 (6.3–14.8)	Median(IQR): 0.2 (0.1–0.3)	138 (52)	Median(IQR): 67 (66–67)
Hwang 2018	ART	Median (IQR):60 (55–65)	50/210/106	T2:98 T3:268	UC	< 0.1	313 (85.8)	Median(IQR): 64.8 (61.2–66.0)
	ESRT	Median (IQR):61 (54.6–65.3)	33/209/124	T2:109 T3:257	UC	Median(IQR): 0.3 (0.2–0.4)	318 (86.9)	Median(IQR): 66.0 (64.8–70.0)
Borghetti 2017	ART	Overall median (range): 65 (42–78)	24/60/43	T2:20 ≥ T3:107	UC	UC	99 (78.0)	UC
	SRT		42/57/32	T2:50 ≥T3:81	UC	UC	80 (61.1)	UC
Hervas 2017	ART	Mean (range):62.7 (43.0–75.0)	199(≤7)/20	≤T2:83 ≥ T3:124	UC	≤0.5	156 (70.9)	UC
	SRT	Mean (range):64.8 (42.0–82.0)	393(≤7)/47	≤T2:272 ≥ T3:172	UC	UC	214 (47.5)	UC
Mishra 2015	ART	Median:59	12/40/19	UC	UC	Median:< 0.1	60 (81.1)	Median:66
	SRT	Median:63	22/49/33	UC	UC	Median:0.6	86 (76.8)	Median:66.6
Hsu 2015	ART	UC	14/34/26	≤T2:12 ≥ T3:64	UC	< 0.1	50 (79)	UC
	SRT	UC	22/118/83	≤T2:86 ≥ T3:143	UC	Median(IQR): 0.5 (0.3–1.0)	149 (86)	UC
Tilki 2016	ART	Median (IQR):65 (60–70)	0/116/97	T2:9 T3:204	Median(IQR): 12 (7.8–25.7)	UC	171 (80.3)	range:60–70
	SRT	Median (IQR):66 (61–70)	1/340/163	T2:63 T3:441	Median(IQR): 11 (7.0–18.9)	UC	212 (42)	range:60–70
Detti 2012	ART	Mean ± SD:65.1 ± 7.3	44/77/82	T2:22 ≥T3:181	Mean ± SD: 0.10 ± 0.28	Mean ± SD: 0.47 ± 1.73	101 (49.8)	Mean ± SD: 66.2 ± 4.1
	SRT	Mean ± SD:67.0 ± 6.0	25/26/53	T2:23 ≥T3:81	Mean ± SD: 0.85 ± 0.52	Mean ± SD: 1.73 ± 3.19	24 (23.1)	Mean ± SD: 66.8 ± 4.1
Ost 2011	ART	Median(range):63 (51–77)	64(including 3 + 4)/25(including 4 + 3)	T2:21 ≥T3:68	Median(range): 10.0 (3.0–47.9)	< 0.2	68 (76)	Median:74
	SRT	Median(range):64 (42–75)	64(including 3 + 4)/25(including 4 + 3)	T2:21 ≥T3:68	Median(range): 10.0 (3.5–148)	≥0.2	59 (66)	Median:76
Trabulsi 2008	ART	Median(range):62.0 (42–76)	22/17/57	≥T3:96	Median(range): 8.3 (1.1–65.9)	< 0.2	80 (83)	Median(range): 60 (50–70)
	SRT	Median(range):63.0 (47–75)	22/17/57	≥T3:96	Median(range): 9.0 (1.7–39)	Median(range): 0.7 (0.2–2)	80 (83)	Median(range): 64.8 (59–70)
Tsien 2003	ART	Median(range):63.0 (43.8–75.7)	11/16/8	≥T3:36	Median(range): 11.6 (1.1–99.6)	UC	34 (89)	Median(range): 64.0 (59.4–69.0)
	SRT	Median(range):64.2 (42.1–78.6)	17/27/8	≥T3:38	Median(range): 13.3 (0.2–120.0)	Median(range): 1.2 (0.2–18.4)	27 (47)	Median(range): 65.0 (60.0–75.0)
Taylor 2003	ART	median:60	9/35/30	≤T2:27 ≥ T3:48	Median:11	Median:< 0.1	73 (96)	Median(range): 60 (51–70)
	SRT	UC	18/27/24	≤T2:12 ≥ T3:59	UC	UC	UC	Median(range): 70 (60–78)
Kalapurakal 2002	ART	Overall median (range): 60 (48–78)	Overall 66(≤7)/10	UC	Overall median(range):12.0 (4–82)	UC	overall 40 (53)	Median(range): 60 (60–65)
	SRT			UC		Median(range): 0.5 (0.2–6.5)		Median(range): 65 (60–70)

UC: unclear

### Overall survival

**Fig. 2:**
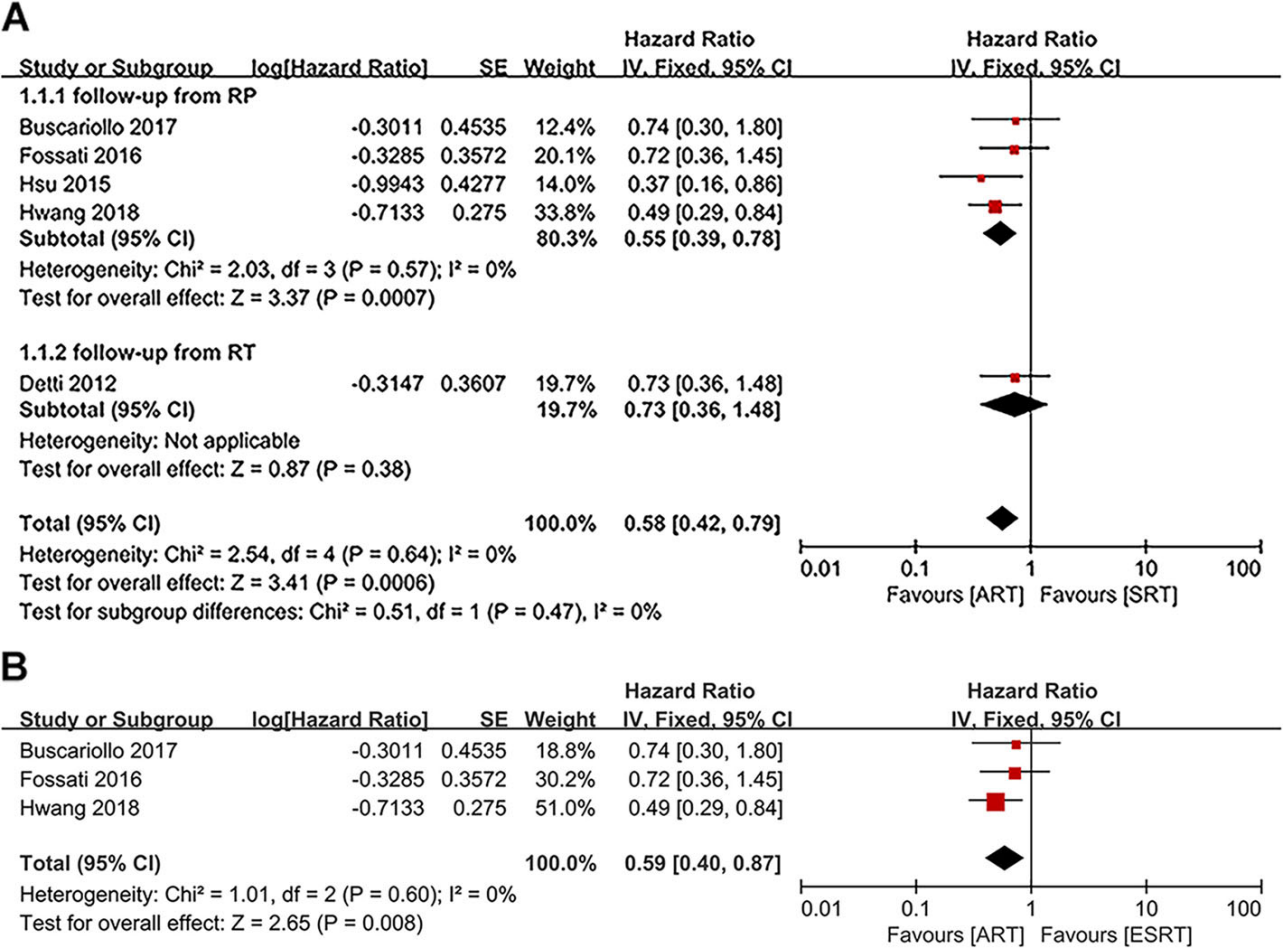
**a**): Forest plot and subgroup analysis in accordance with the starting point of follow-up of OS. **b**): Forest plot of OS, when comparing patients receiving ART and ESRT after radical prostatectomy

The HRs and corresponding 95% CIs of OS in five articles were pooled and the results showed that OS in the ART group was better than that in the SRT group (HR: 0.58; 95%CI: 0.42–0.79; *p* = 0.0006) with no statistically significant heterogeneity (I^2^ = 0, *p* = 0.64) [15–17, 21, 23] (Fig. 2a). Subgroup analysis also indicated a statistically significant difference between postoperative patients received ART and SRT when follow-up time was calculated from RP (HR: 0.55; 95%CI: 0.39–0.78; *p* = 0.0007). No significant heterogeneity was found among studies (I^2^ = 0, *p* = 0.57). However, the subgroup analysis of follow-up from RT was not applicable (Fig. 2a).

We also compared the OS of patients who received post-prostatectomy ART versus ESRT, which were extracted from three studies [15–17]. The meta-analysis of these studies showed that ART still had an advantage over ESRT in terms of OS (HR:0.59; 95%CI: 0.40–0.87; *p* = 0.008). Likewise, there was no evidence to reveal a significant heterogeneity between studies (I^2^ = 0, *p* = 0.60) (Fig. 2B).

**Fig. 3:**
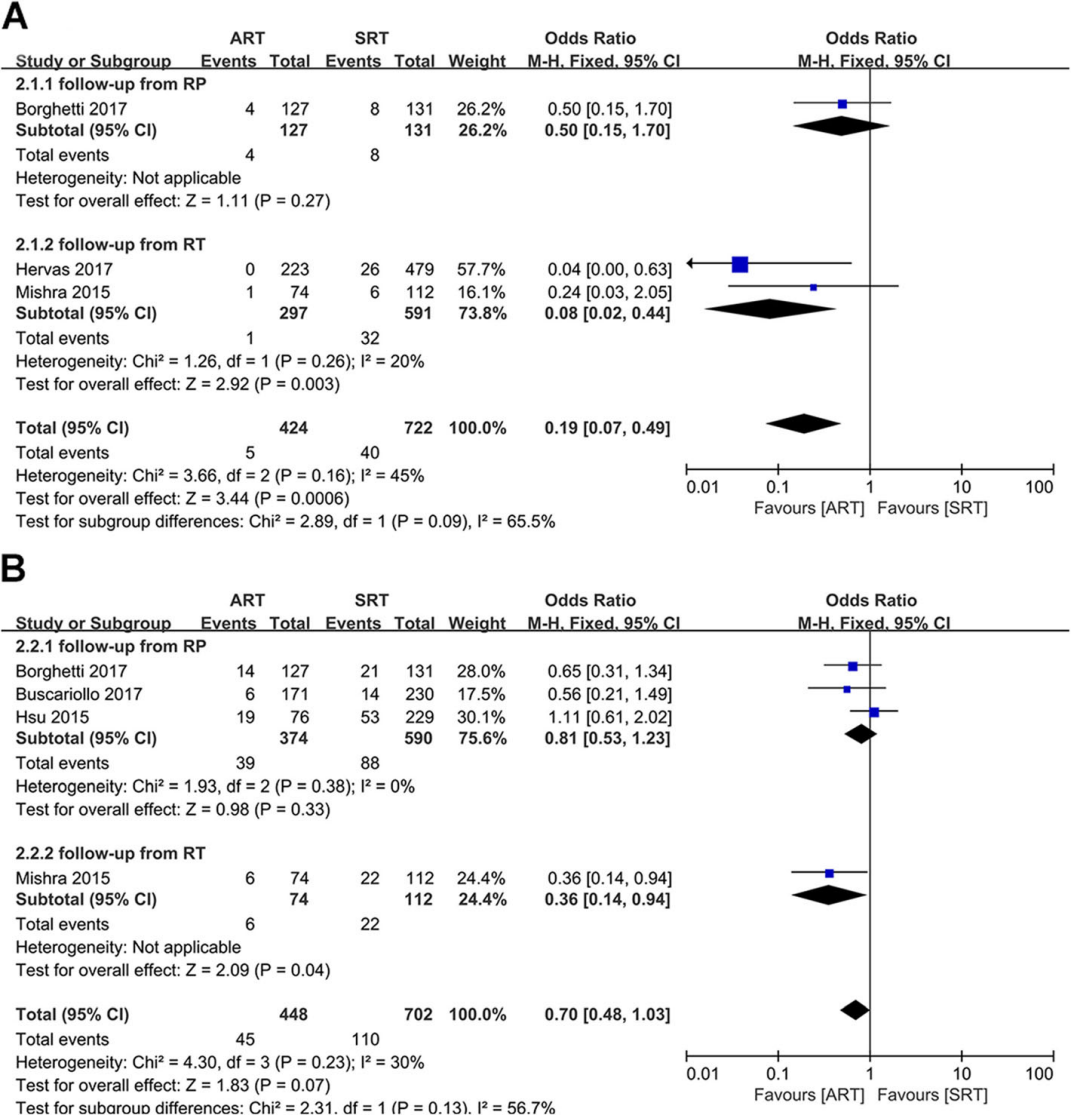
**a**): Forest plot and subgroup analysis in accordance with the starting point of follow-up of 5-year OS rate. **b**): Forest plot and subgroup analysis in accordance with the starting point of follow-up of 10-year OS rate

Three studies reported 5-year OS rates of the ART and SRT groups [18–20], and four studies reported 10-year OS rates of these patients [15, 18, 20, 21]. The pooled results of 5-year OS rate showed there was a significant statistical difference between the ART group and the SRT group (OR: 0.19; 95%CI: 0.07–0.49; *p* = 0.0006; I^2^ = 45%) (Fig. 3a). Similarly, subgroup analysis presented a statistically significant difference between the ART group and the SRT group when follow-up from RT (OR: 0.08; 95%CI: 0.02–0.44; *p* = 0.003; I^2^ = 20%) (Fig. 3a). But the subgroup analysis of follow-up from RP was not applicable (Fig. 3a).

Nevertheless, the pooled result of 10-year OS rate suggested there was no significant statistical difference between the ART group and the SRT group (OR:0.70; 95%CI: 0.48–1.03; *p* = 0.07; I^2^ = 30%) (Fig. 3B). Subgroup analysis indicated that there was also no statistically significant difference between two groups in terms of 10-year OS rate for studies calculated from RP (OR: 0.81; 95%CI: 0.53–1.23; *p* = 0.33; I^2^ = 0%) (Fig. 3b). The subgroup analysis of follow-up from RT was also not applicable (Fig. 3b).

### Biochemical recurrence-free survival

**Fig. 4:**
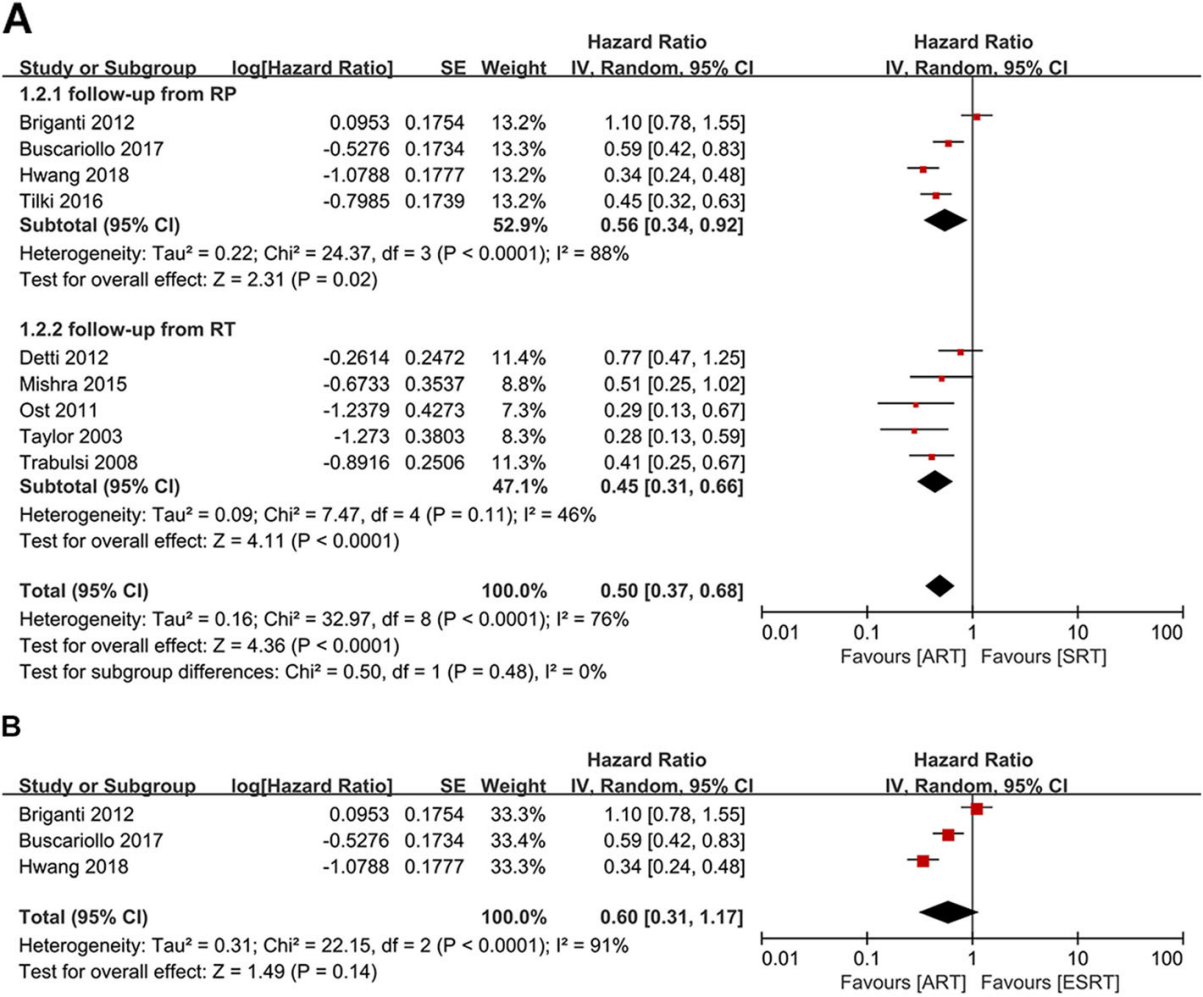
**a**): Forest plot and subgroup analysis in accordance with the starting point of follow-up of BRFS. **b**): Forest plot of BRFS, when comparing patients receiving ART and ESRT after radical prostatectomy

The HRs and corresponding 95% CIs of BRFS between the ART and SRT groups were extracted from nine articles [14, 15, 17, 20, 22–25, 27]. A meta-analysis of these studies by a random effect model showed that postoperative patients who received ART had better control of BCR compared to those received SRT (HR: 0.50; 95%CI: 0.37–0.68; *p* < 0.0001) with a statistically significant heterogeneity (I^2^ = 76%, *p* < 0.0001) (Fig. 4a). The pooled result of data reported in four studies [14, 15, 17, 22], which took RP as the starting point of follow-up, showed there was a significant statistical difference between the ART and the SRT groups (HR: 0.56; 95%CI: 0.34–0.92; *p* = 0.02) with a statistically significant heterogeneity (I^2^ = 88%, p < 0.0001) (Fig. 4a). The subgroup analysis of follow-up from RT revealed the similar tendency that the ART group owned a longer BCR process (HR: 0.45; 95%CI: 0.31–0.66; p < 0.0001) (Fig. 4a). No statistically significant heterogeneity was found among these studies (I^2^ = 46%, *p* = 0.11) (Fig. 4a).

In the same way, we compared the BRFS of patients who received post-prostatectomy ART versus ESRT, which were obtained from three studies [14, 15, 17]. Conversely, the meta-analysis of these studies illustrated there was no statistical difference in BRFS between the ART and ESRT groups (HR: 0.60; 95%CI: 0.31–1.17; *p* = 0.14; I^2^ = 91%). (Fig. 4b).

**Fig. 5:**
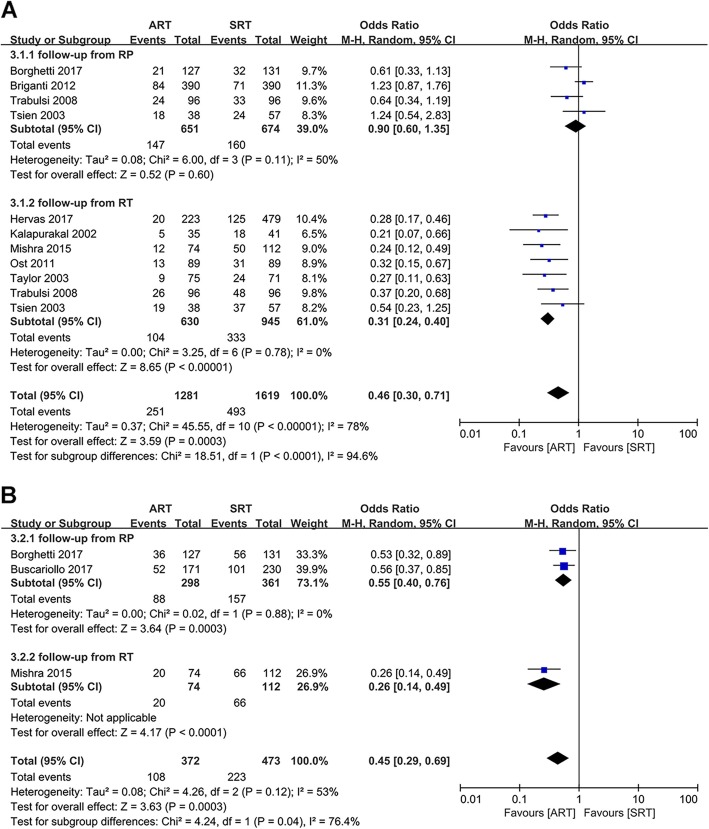
**a**): Forest plot and subgroup analysis in accordance with the starting point of follow-up of 5-year BRFS rate. **b**): Forest plot and subgroup analysis in accordance with the starting point of follow-up of 10-year BRFS rate

The 5-year BRFS rate of ART and SRT groups were reported in nine studies [14, 18–20, 24–28]. Data were pooled with a random-effect model since there was a significant difference in heterogeneity among these studies (I^2^ = 78%, *p* < 0.00001). The pooled result of the 5-year BRFS rate showed there was a significant statistical difference between the ART and the SRT groups (OR: 0.46; 95%CI: 0.30–0.71; *p* = 0.0003) (Fig. 5a). Of these studies, two calculated BRFS from the time of RP, five calculated BRFS from the time of RT and the remaining two reported 5-year BRFS rate both at these two different time points. The subgroup analysis showed 5-year BRFS rate between the ART and SRT groups were comparable (OR: 0.90; 95% CI: 0.60–1.35; *p* = 0.60) with no statistically significant heterogeneity (I^2^ = 50%, *p* = 0.11) when follow-up started from RP (Fig. 5a). However, the pooled data followed up from RT indicated that there was a significant statistical difference between the ART and the SRT groups (OR: 0.31; 95%CI: 0.24–0.40; *p* < 0.00001), which was consistent with the overall outcome (Fig. 5a). No significant heterogeneity was found among studies (I^2^ = 0, *p* = 0.78) (Fig. 5a).

Three studies reported 10-year BRFS rate of these patients [15, 18, 20]. Random effect model meta-analysis was conducted since there was a significant difference in heterogeneity among these studies (I^2^ = 53%; *p* = 0.12). The pooled analysis showed that there was a significant statistical difference between the ART and the SRT groups (OR: 0.45; 95%CI: 0.29–0.69; *p* = 0.0003) (Fig. 5b). The subgroup analysis of follow-up started from RP indicated that there was also a significant statistical difference in 10-year BRFS rate between the ART and the SRT groups (OR: 0.55; 95% CI: 0.40–0.76; p = 0.0003) with no statistically significant heterogeneity (I^2^ = 0, *p* = 0.88) (Fig. 5b).

### Distant metastases-free survival

**Fig. 6:**
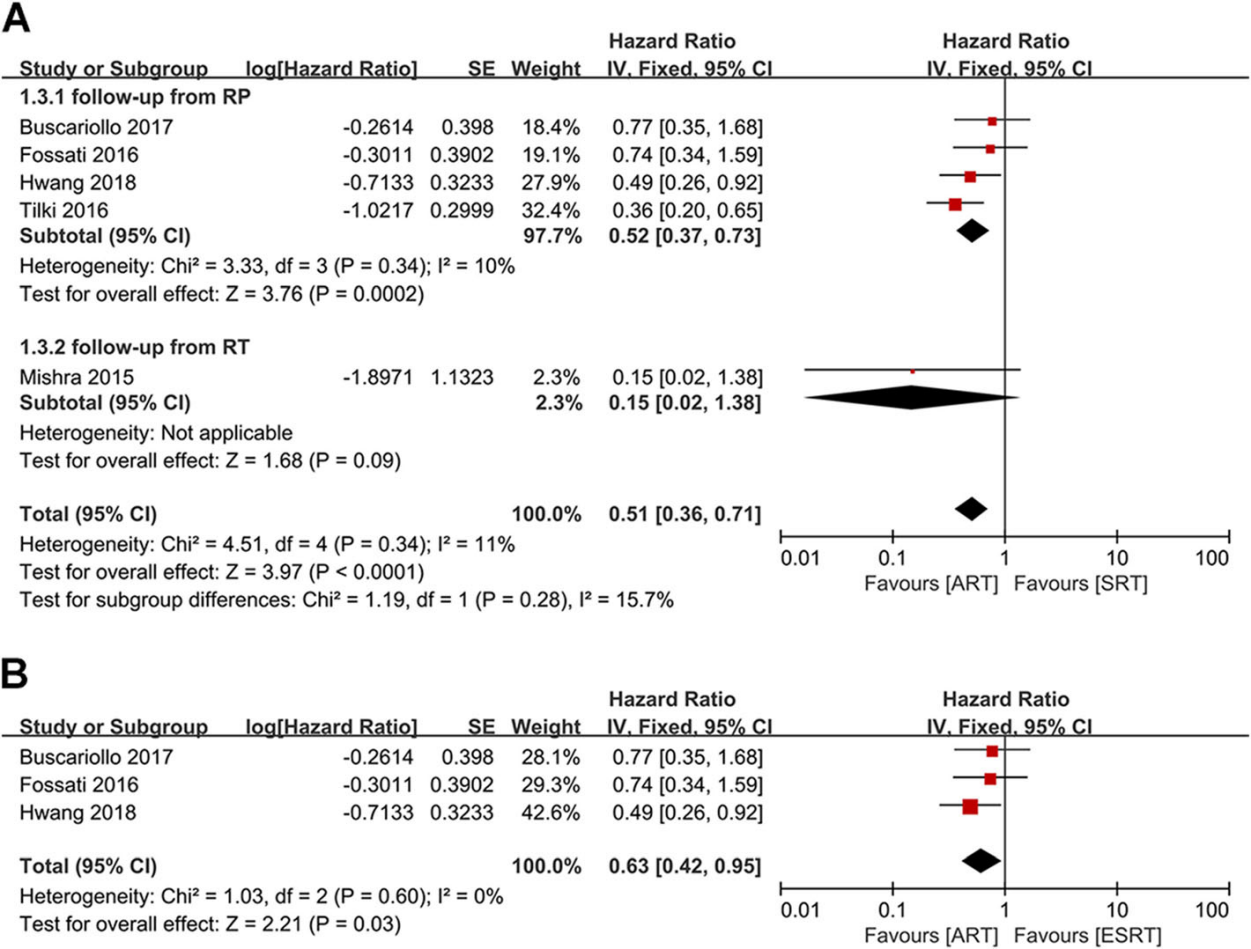
**a**): Forest plot and subgroup analysis in accordance with the starting point of follow-up of DMFS. **b**): Forest plot of DMFS, when comparing patients receiving ART and ESRT after radical prostatectomy

The HRs and corresponding 95% CIs of DMFS were available in five studies [15–17, 20, 22]. A meta-analysis of these studies by a fixed effect model demonstrated that patients in the ART group had a lower risk of distant metastasis than those in the SRT group (HR: 0.51; 95%CI: 0.36–0.71; *p* < 0.0001) with no statistically significant heterogeneity (I^2^ = 11%, *p* = 0.34) (Fig. 6a). The subgroup analysis also produced a similar result that there was significant statistical difference in DMFS between the ART and the SRT groups (HR: 0.52; 95%CI: 0.37–0.73; *p* = 0.0002) without statistically significant heterogeneity (I^2^ = 10%, p = 0.34) when follow-up from RP in four studies [15–17, 22] (Fig. 6a).

When compared the data from three studies focused on DMFS of patients who treated with post-prostatectomy ART versus ESRT [15–17], the pooled analysis showed that there was a significant statistical difference between the ART and the ESRT groups (HR: 0.63; 95% CI: 0.42–0.95; *p* = 0.03). No significant heterogeneity was found among studies (I^2^ = 0, *p* = 0.6) (Fig. 6b).

### Quality assessment and publication bias

**Fig. 7:**
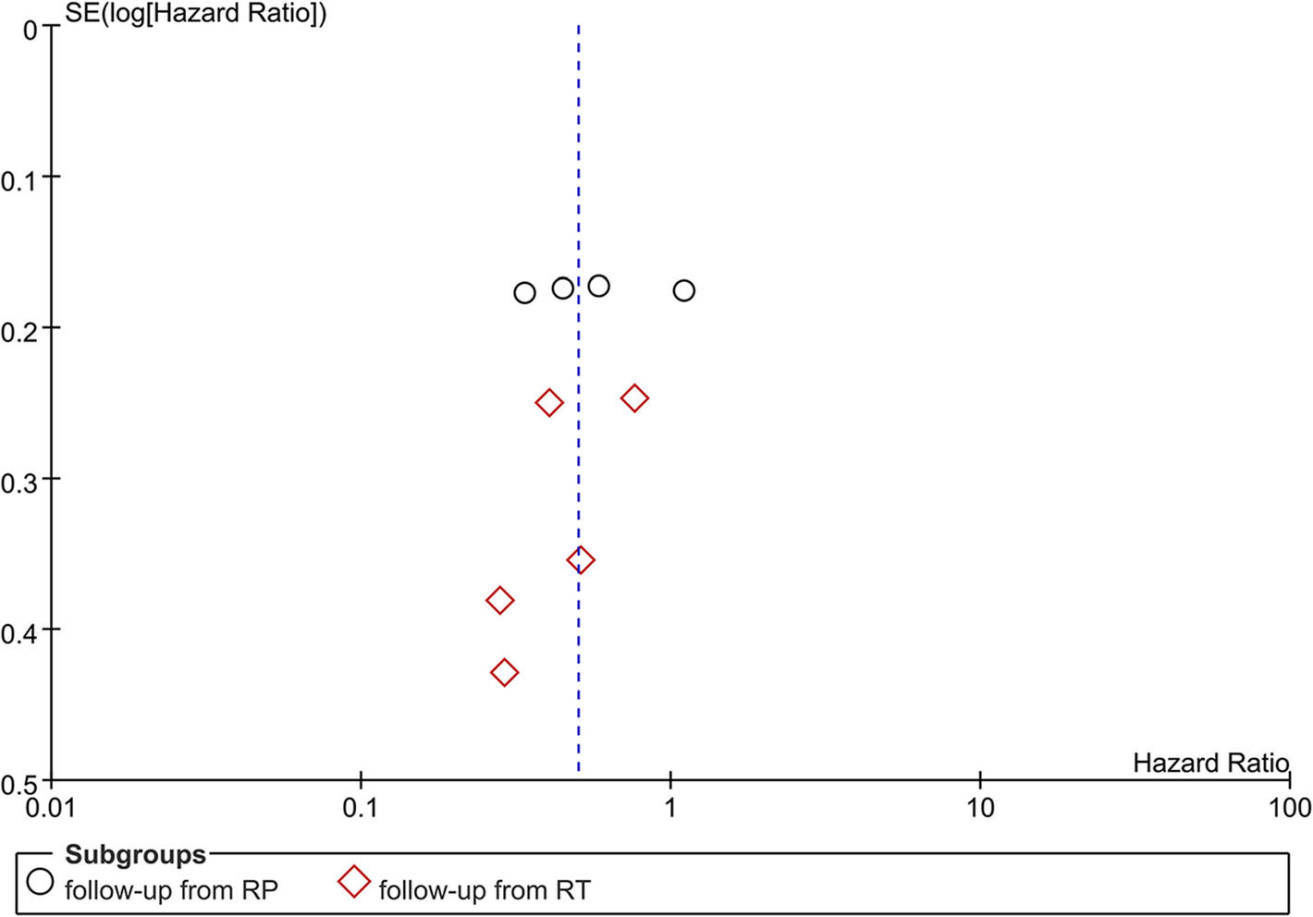
Funnel plot constructed by biochemical recurrence-free survival (BRFS) of the included studies compared ART with SRT

The NOS scores of included studies ranged from 5 to 8 (median score: 7), which meant that all references rated at least moderate quality. The distribution of NOS scores of all included studies in this meta-analysis was presented in Table 3. A funnel plot of BRFS was made to evaluate the publication bias. No significant asymmetry can be found and it suggested there was no significant publication bias in this study **(**Fig. 7**)**.

**Table 3:** Quality assessment of included studies

Study	Selection	Comparability	Outcome	Total	Quality level
Briganti 2012	3	2	3	8	High
Buscariollo 2017	3	2	3	8	High
Fossati 2016	3	1	3	7	Moderate
Hwang 2018	3	2	2	7	Moderate
Borghetti 2017	3	1	2	6	Moderate
Hervas 2017	3	0	3	6	Moderate
Mishra 2015	3	2	2	7	Moderate
Hsu 2015	3	2	3	8	High
Tilki 2016	3	2	3	8	High
Detti 2012	3	1	3	7	Moderate
Ost 2011	3	2	2	7	Moderate
Trabulsi 2008	3	2	2	7	Moderate
Tsien 2003	3	1	2	6	Moderate
Taylor 2003	3	1	2	6	Moderate
Kalapurakal 2002	3	1	1	5	Moderate

## Discussion

As a matter of fact, 17–64% of patients who undergo RP would appear BCR, and up to one-third of men with BCR would develop metastatic diseases and eventually die of PCa [36]. Under these circumstances, the important role of postoperative radiotherapy is self-evident. According to the consensus reached by the American Urological Association (AUA) and the American Society of Radiation Oncology (ASTRO), patients with APFs should be informed that ART, compared to RP only, could reduce the risk of BCR, local recurrence, and clinical progression of cancer [4]. It also states that physicians should offer SRT to patients with BCR or local recurrence after RP, but without distant metastases [37]. So far, however, no definitive conclusion has been reached regarding the survival benefits of optimal timing of RT for patients with APFs following RP.

This systematic review and meta-analysis were designed to assist clinicians and patients to make optimal decisions by comparing the effect of ART and SRT on prognosis after RP. The pooled results of OS, BRFS, and DMFS revealed that ART could obtain better control of PCa disease and improve the survival outcomes when compared to SRT. ART also had advantages over SRT in both 5- and 10-year BCR rate. The analysis of 5-year OS rate demonstrated that ART still had survival advantages compared to SRT. However, ART and SRT were similar in 10-year OS rate. The loss of follow-up and censored data of these postoperative patients might account for these outcomes. Furthermore, with the development of PCa, some patients who received SRT might be also treated with ADT, which would undoubtedly improve the efficacy of SRT. To sum up, it seems more advisable for patients with APFs after RP to receive ART to avoid missing the appropriate timing of radiotherapy.

Additionally, wait-and-see along with delayed RT until PSA starts to rise for postoperative patients with negative PSA could spare partial individuals from receiving unnecessary treatment since they might not develop a clinical recurrence. However, Oort et al. reported that GS, pathologic stage, and PSM of RP specimens were the most powerful predictors of disease progression [38]. Swanson et al. also showed that positive seminal vesicles, Gleason sum score 8–10, extracapsular extension, and PSM were highly strong predictor of failure after prostatectomy [39]. Hence, there is no deny that the above prognostic factors must be considered comprehensively for clinicians and radiologists when planning ART for postoperative patients.

In fact, there is an ongoing RCT to compare outcomes of ART and ESRT, namely the radiotherapy assisted treatment and early rescue (RAVES) trial, which is led by the Trans Tasman Radiation Oncology Group (TROG), in collaboration with the Urological Society of Australia and New Zealand (USANZ), and the Australian and New Zealand Urogenital and Prostate Cancer Trials Group (ANZUP) [40]. Because of the specificity of prostate cancer progression, the results of this clinical trial might take a long time to be known. In order to get more comprehensive results, we also specifically analyzed the retrospective information about the outcomes of ART and ESRT in this meta-analysis. It suggested that ART was superior to ESRT on OS and DMFS, and similar to ESRT on BRFS.

In our review, the limitations that we should discuss are as follows. First, the information was obtained from a number of retrospective studies, which might introduce confounding data and produce bias. Second, all studies were carried out between 1987 and 2013 in different countries. The development of RT and the different implementation standards of RT in different regions could affect the prognosis of patients after RP. Main types of RT used to treat PCa including external beam radiation therapy and brachytherapy. Even patients from the same region would receive RT in different ways, which might affect the final results. In addition, most of the included studies paid more attention to biochemical control or OS of postoperative patients treated with ART and SRT and rarely reported adverse effects caused by these two radiotherapies. Finally, some subjects in the included studies received RT along with ADT, which might affect the differences between the two types of radiation.

## Conclusion

According to this meta-analysis, ART was superior to SRT (including ESRT) on OS and DMFS and could be served as a preferential treatment for patients with APFs after RP to achieve a better prognosis. Certainly, high-quality, multicenter RCTs are expecting to confirm the outcomes of our meta-analysis in the future.

## Data Availability

All data generated or analyzed during this study are included in this manuscript.

## Abbreviations

ADT: Androgen-deprivation therapy
APFs: Adverse pathological features
ART: Adjuvant radiotherapy
BCR: Biochemical recurrence
BRFS: Biochemical recurrence-free survival
CI: Confidence interval
DMFS: Distant metastases-free survival
ESRT: Early salvage radiotherapy
GS: Gleason score
HR: Hazard ratio
NOS: Newcastle-Ottawa scale
OR: Odds ratio
OS: Overall survival
PCa: Prostate cancer
PSA: Prostate-specific antigen
PSM: Positive surgical margins
RCTs: Randomized controlled trials
RP: Radical prostatectomy
RT: Radiotherapy
SRT: Salvage radiotherapy
